# Supplementary material for: RT-qPCR Detection of SARS-CoV-2: No Need for a Dedicated Reverse Transcription Step
Source: Int J Mol Sci. 2022 Jan 24;23(3):1303. doi: 10.3390/ijms23031303 (PMC8835954; doi:10.3390/ijms23031303)
Supplement: Supplementary file 1 [file ijms-23-01303-s001.zip › ijms-1520356-Supplementary Figures.pdf]

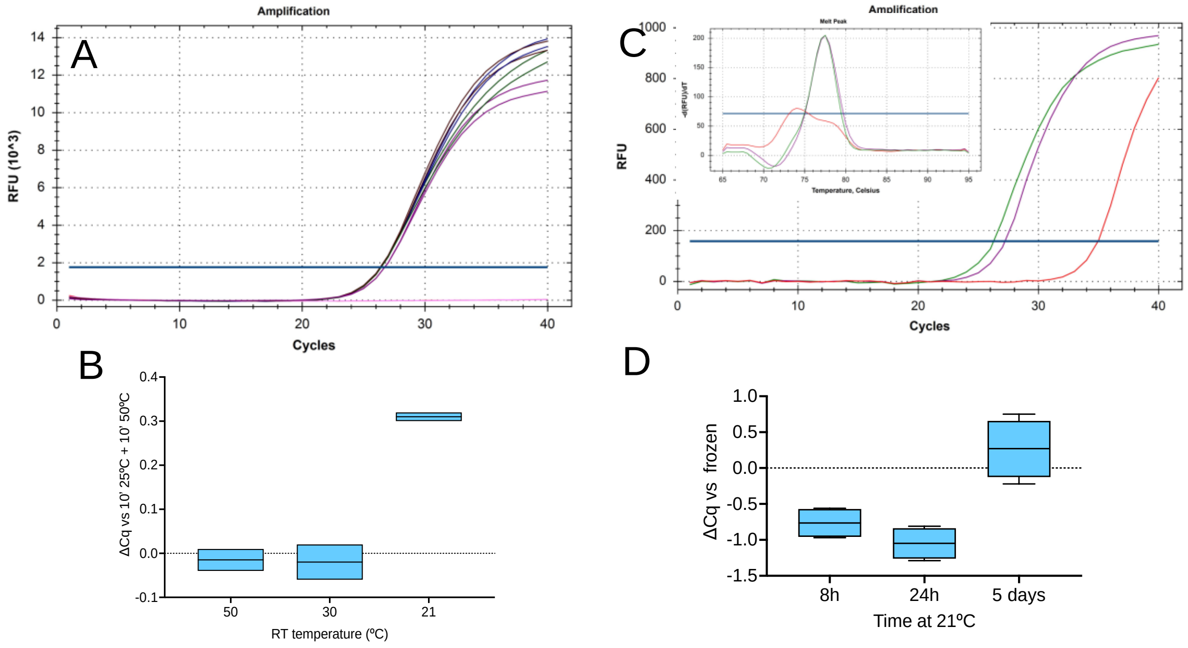


**Figure S1.** Amplification of RNA reverse transcribed by SS4, amplified using primers targeting the viral N-gene (assay N1) and detected using a hydrolysis probe or SYBR Green. **A.** Amplification plots (probe) and Cqs from duplicate qPCR reactions: 10 minutes at 25ºC and 50ºC (protocol i, blue), 5 minutes at 50ºC (protocol ii, green), 30ºC (protocol iii, brown) or 21ºC (protocol iv, purple). **B.** Box and whiskers plot showing the ΔCq for the three modified conditions relative to the recommended protocol, with the whiskers delineating the minimum and maximum values. **C.** Amplification plots (SYBR Green) and melt curves (inset) from single qPCR reactions: 5 minutes at 50ºC (protocol ii, green) or 21ºC (protocol iv, purple). The NTC is shown in red. **D.** Cq values of RNA samples left on the bench for the indicated times relative to control sample kept frozen at -80ºC.

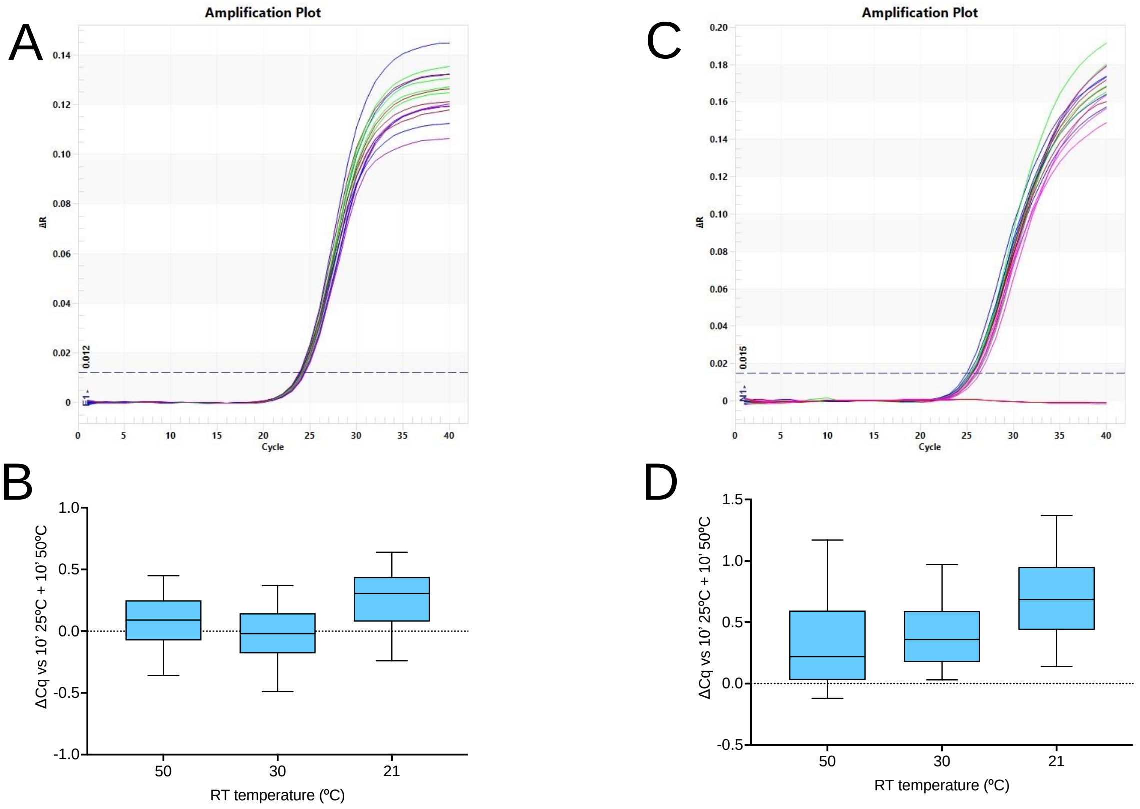


**Figure S2.** Amplification on the PCRMax Eco instrument of RNA reverse transcribed by SS4, amplified using primers targeting the viral E (assay E1) and N-genes (assay N1) and detected using a hydrolysis probe. **A.** Amplification plots (probe) and Cqs from qPCR reactions targeting E1: 10 minutes at 25ºC and 50ºC (protocol i, blue), 5 minutes at 50ºC (protocol ii, green), 30ºC (protocol iii, brown) or 21ºC (protocol iv, purple). **B.** Box and whiskers plot showing the ΔCq for the three modified conditions relative to the recommended protocol, with the whiskers delineating the minimum and maximum values. **C.** Amplification plots (probe) and Cqs from qPCR reactions targeting N1: 10 minutes at 25ºC and 50ºC (protocol i, blue), 5 minutes at 50ºC (protocol ii, green), 30ºC (protocol iii, brown) or 21ºC (protocol iv, purple). **B.** Box and whiskers plot showing the ΔCq for the three modified conditions relative to the recommended protocol, with the whiskers delineating the minimum and maximum values.

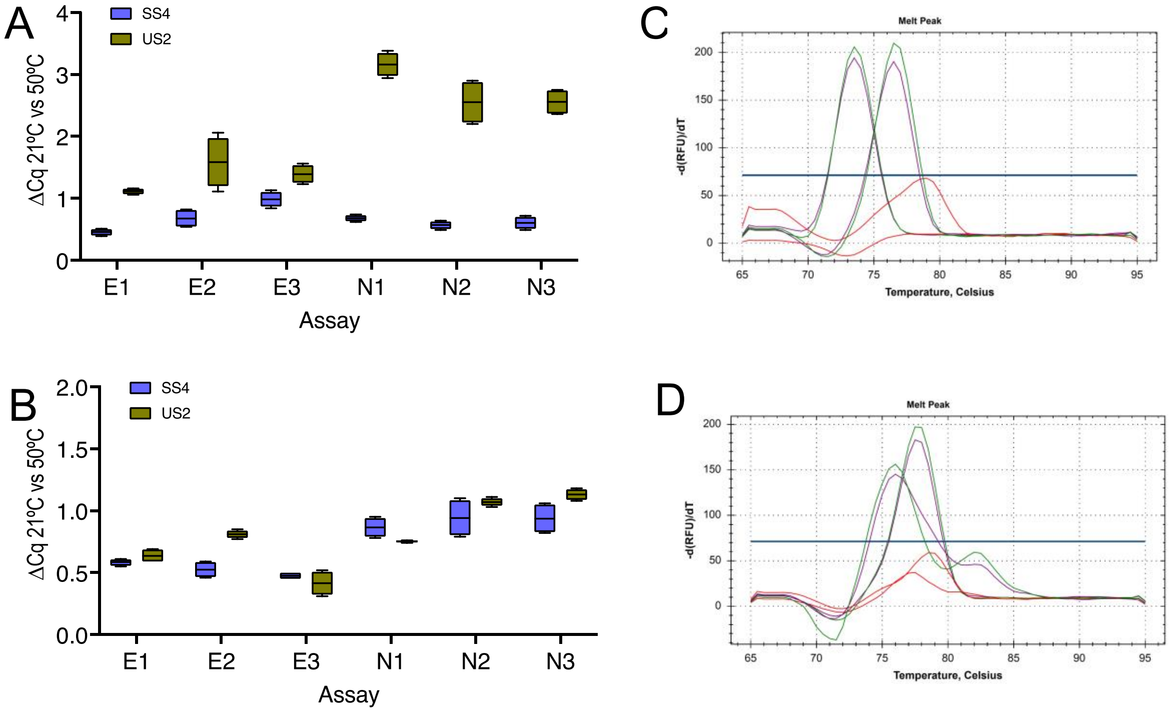


**Figure S3.** Comparison of two further RNA sample reverse transcribed by SS4 and US2 amplified using assays E1 to E3 and N1 to N3. **A.** Box and whiskers plot comparing the ΔCq (21ºC vs 50ºC) for the six assays with SS4 (blue) and US2 (olive) cDNAs, respectively, carried out with the first RNA sample. **B.** The same plot carried out with the results recorded for the second RNA sample. **C.** Melt curves for E2 and E3 PCR amplicons recorded from RT reactions carried out at 50ºC (brown) or 21ºC (green). The red melt profiles were obtained from the NTC controls. **D.** Melt curves for N2 and N3 PCR amplicons recorded from RT reactions carried out at 50ºC (brown) or 21ºC (green). The red melt profiles were obtained from the NTC controls.

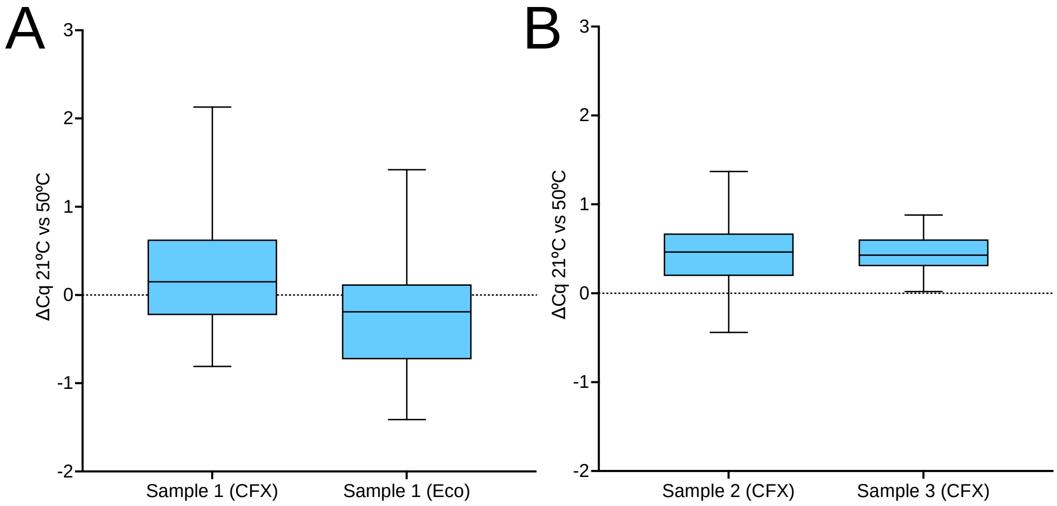


**Figure S4.** Reproducibility of low temperature RT relative to the standard 5-minute 50ºC protocol and amplified with the E1 assay. **A.** Aliquots of the same sample were transcribed by SS4 at 50ºC or 21ºC for 5 minutes and amplified on CFX or Eco instruments. **B.** RT reactions were repeated with a second and third RNA sample transcribed by SS4 at 50ºC or 21ºC for 5 minutes and amplified on the CFX. All box and whiskers plots show the ΔCq for the reactions carried out at 21ºC relative to 50ºC, with the whiskers delineating the minimum and maximum ΔCq values. The scales of the y-axes were adjusted to a common scale to simplify the comparison between the runs and instruments.

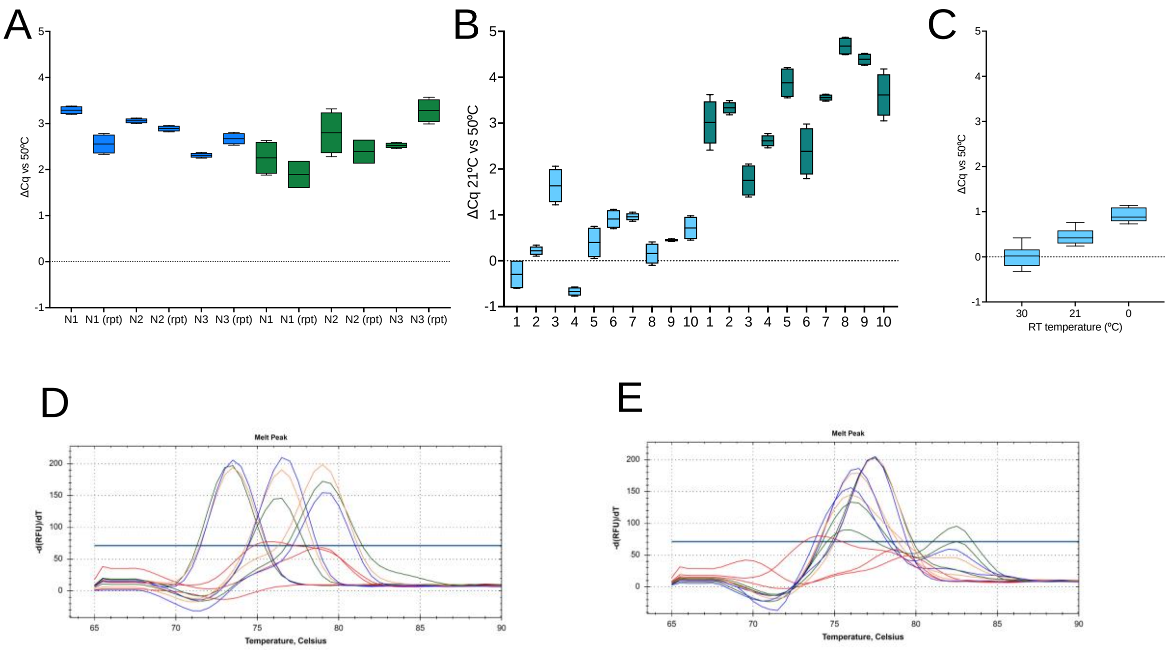


**Figure S5.** RT efficiency at 0ºC. **A.** ΔCqs against control RNA transcribed at 50ºC of RNA transcribed in duplicate on ice by SS4 (blue) or US2 (green) and amplified with assays N1, N2 and N3. **B.** ΔCqs against control RNA transcribed at 50ºC of RNA transcribed in ten independent RT reactions for 5 minutes at 21ºC (blue) or ice (green) with US2 and amplified with assay E1 on the CFX. **C.** ΔCqs against control RNA transcribed at 50ºC of RNA transcribed for 5 minutes at 30ºC, 21ºC or ice with SS4 and amplified with assay E1 on the CFX **D.** Melt curves of PCR amplicons for assays E1, E2 and E3 obtained at 30ºC (orange), 21ºC (blue) and 4ºC (green). The red melt curve is from the NTC. **E.** Melt curves of PCR amplicons for assays N1, N2 and N3 obtained at 30ºC (orange), 21ºC (blue) and 4ºC (green). The red melt curve is from the NTC. All box and whiskers plots show the ΔCq for the reactions carried out at 21ºC relative to 50ºC, with the whiskers delineating the minimum and maximum ΔCq values. The scales of the y-axes were adjusted to a common scale to simplify the comparison between the runs and instruments.

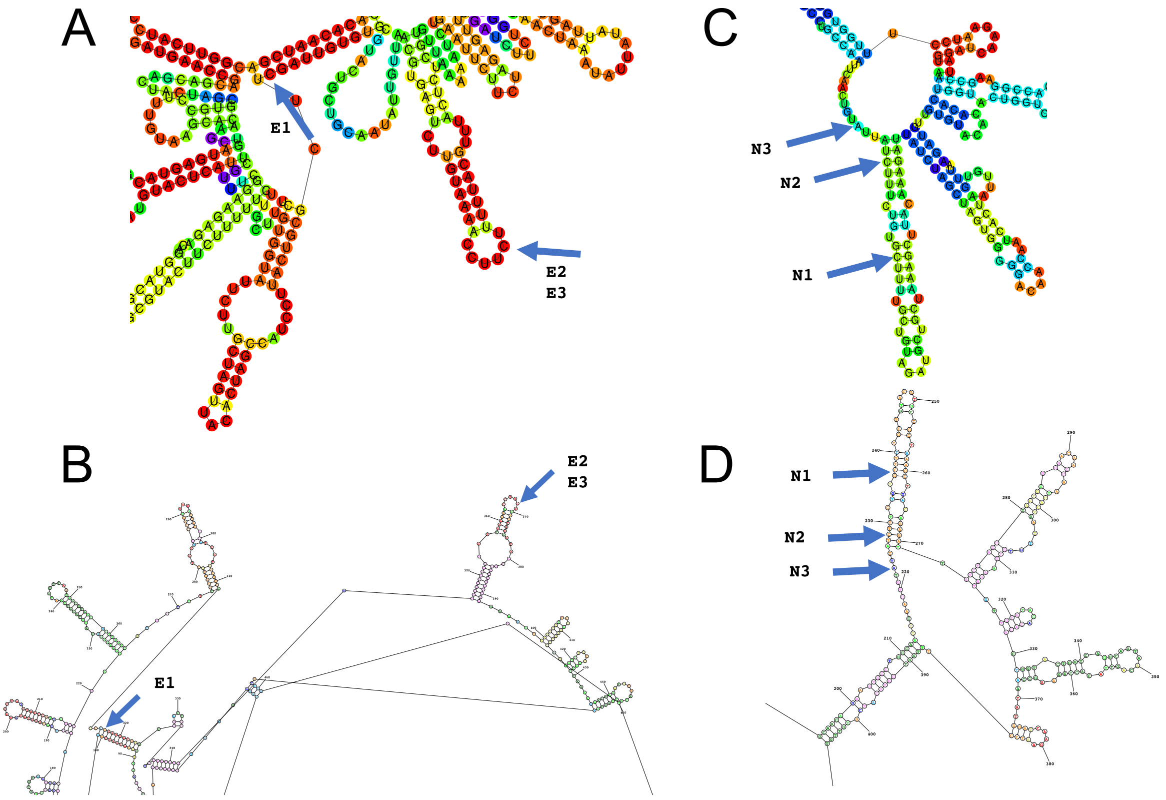


**Figure S6.** Mapping of reverse primers to SARS-CoV-2 secondary and tertiary genomic RNA structure. **A.** The locations of primers initiating reverse transcription of assays E1, E2 and E3 are indicated by arrows on one of the predicted structures [43]. The E1 primer maps into a stem structure, whereas the 3′-ends of both E2 and E3 primers terminate in a small loop, **B.** The second model [44] results in a similar prediction for the three primers. **C.** The locations of primers initiating reverse transcription of assays N1, N2 and N3 are indicated by arrows on one of the predicted structures [43]. N1 and N2 primers map into a stem structure, whereas the 3′-end of the N3 primer terminates in a small loop, **D.** The second model [44] results in a similar prediction for the three primers.

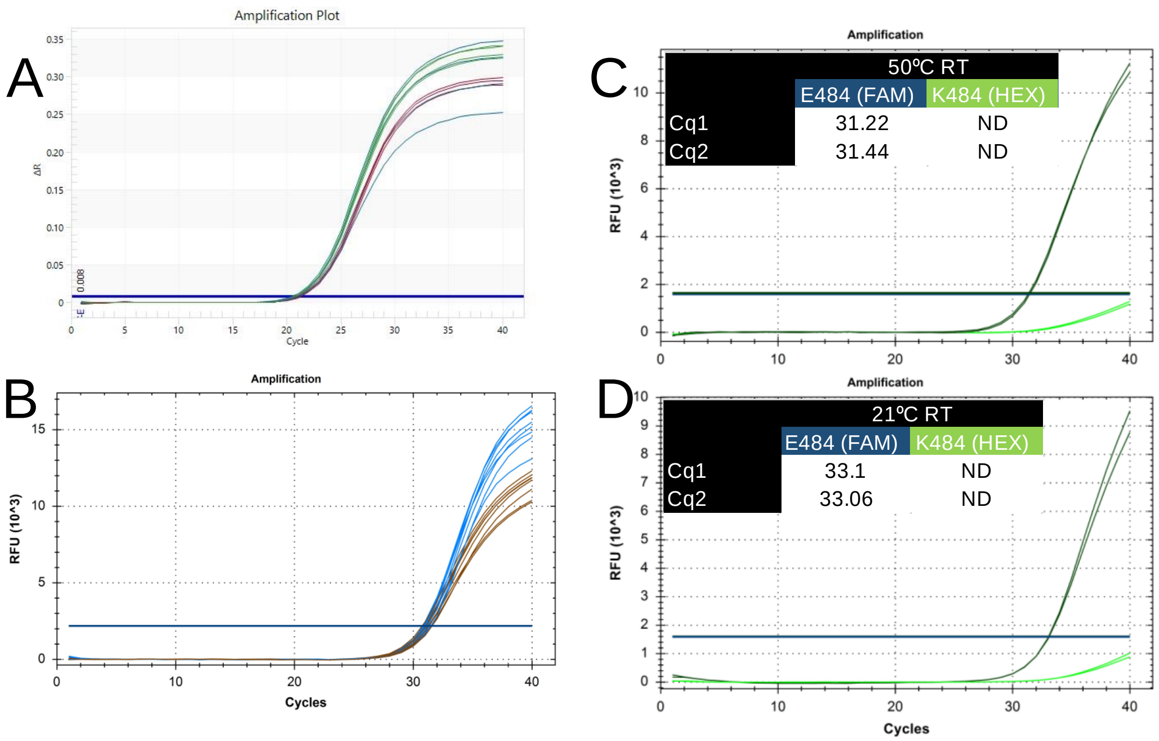


**Figure S7.** RT reactions carried out with high and low copy number targets. **A.** Two-step assay (SS4/Bioline SensiFast) with E1 and a high viral load sample. RT reactions were carried out at 50ºC (blue), 30ºC (green) and 21ºC (brown). **B.** One step gradient RT-qPCR (PCRBio 1-step) with E1 (blue) and N1 (brown) and a low viral load. **C.** One-step RT-qPCR (PS3) detection of the WT E484 sequence in variant B.1.1.7 with a 50ºC RT reaction. **D.** One-step RT-qPCR (PS3) detection of the WT E484 sequence in variant B.1.1.7 with a 21ºC RT reaction.
